# Antimicrobial Resistance Trends in Hidradenitis Suppurativa Lesions

**DOI:** 10.3390/jcm13144246

**Published:** 2024-07-20

**Authors:** Dimitra Koumaki, Georgios Evangelou, Sofia Maraki, Evangelia Rovithi, Danae Petrou, Erato Solia Apokidou, Stamatios Gregoriou, Vasiliki Koumaki, Petros Ioannou, Kyriaki Zografaki, Aikaterini Doxastaki, Alexander Katoulis, Kalliopi Papadopoulou, Dimitra Stafylaki, Viktoria Eirini Mavromanolaki, Konstantinos Krasagakis

**Affiliations:** 1 Dermatology Department, University Hospital of Heraklion, Heraklion, 711 10 Crete, Greece; gevangelou@pagni.gr (G.E.); eva.rovithi@pagni.gr (E.R.); dpetrou@pagni.gr (D.P.); kikizog@yahoo.gr (K.Z.); katerina.doxastaki@gmail.com (A.D.); krasagak@uoc.gr (K.K.); 2 Department of Clinical Microbiology, University Hospital of Heraklion, Heraklion, 711 10 Crete, Greece; smaraki@pagni.gr (S.M.); dstafylaki@pagni.gr (D.S.); 3 Department of Internal Medicine, Agios Nikolaos General Hospital, Knosou 4, Ag. Nikolaos, 721 00 Crete, Greece; esoli@auth.gr; 4 1st Department of Dermatology and Venereology, Medical School of Athens, National and Kapodistrian University of Athens, Andreas Sygros Hospital, I. Dragoumi 5, 161 21 Athens, Greece; stamgreg@med.uoa.gr; 5 Department of Medical Microbiology, Medical School of Athens, National and Kapodistrian University of Athens, 75 Mikras Asias Str., Goudi, 115 27 Athens, Greece; vkoumaki@med.uoa.gr; 6 Department of Internal Medicine, University Hospital of Heraklion, Heraklion, 711 10 Crete, Greece; 7 School of Medicine, University of Crete, 700 03 Iraklio, Greece; 8 2nd Department of Dermatology and Venereology, Medical School, National and Kapodistrian University of Athens, “Attikon” General University Hospital, Rimini 1, Haidari, 124 62 Athens, Greece; akatoulis@med.uoa.gr; 9 2nd Department of Internal Medicine, General Hospital of Venizeleio, Knossou Avenue 44, 71 409 Heraklion, Greece; med3612@edu.med.uoc.gr; 10 Department of Paediatrics, Agios Nikolaos General Hospital, Agios Nikolaos, 721 00 Crete, Greece; med3548@edu.med.uoc.gr

**Keywords:** bacteria, hidradenitis suppurativa, antibiotic resistance, *Staphylococcus lugdunensis*, *Staphylococcus aureus*, penicillin

## Abstract

**Background/Objectives**: Antibiotic (AB) therapy is the first step in managing hidradenitis suppurativa (HS). Knowledge of the local patterns of antimicrobial resistance is paramount for the appropriate selection of antimicrobials. This study aimed to assess the occurrence of antibiotic resistance in patients with HS. **Methods:** A cross-sectional study was conducted on 103 patients with HS seen at the Dermatology Department at the University Hospital of Heraklion, Heraklion, Crete, Greece, from January 2019 to December 2023, who were not on any antibiotics in the last three months. **Results:** A total of 103 patients with HS participated in this study. Purulent material from 139 skin lesions of these patients was swabbed, and 79.86% (111/139) tested positive for bacteria. Gram-positive isolates accounted for 73%, whereas Gram-negative isolates comprised 27%. Among the isolates, 85.1% were aerobes, and 14.9% were anaerobic. The most common bacterial families isolated were *Staphylococcaceae* (48.27%), *Enterobacteriaceae* (14.94%), and *Streptococcaceae* (6.89%). The antibiogram profiles of bacterial cultures revealed a 57.1% resistance to levofloxacin and a 53.3% resistance to penicillin in *Staphylococcus lugdunensis*, whereas *Staphylococcus aureus* showed a 76.9% resistance to penicillin and a 58.3% resistance to fusidic acid. High resistance rates of 63.5% for tigecycline, 63.3% for ampicillin, and 40.5% for colistin were observed for Gram-negative isolates. Resistances of 62.5%, 61.5%, and 53.8% to erythromycin, clindamycin, and penicillin, respectively, were observed in the anaerobes. **Conclusions:** Patients with HS displayed considerable resistance to bacterial proliferation. The revised therapeutic guidelines for HS should incorporate the latest insights into bacterial antibiotic resistance.

## 1. Introduction

Hidradenitis suppurativa (HS) is a chronic inflammatory condition marked by recurrent painful nodules, abscesses, and sinus tracts in areas like the axillae, groin, and perianal region [1,2,3]. This condition significantly impacts patients’ quality of life, highlighting the need for effective management strategies [4,5].

The exact cause of HS is not fully understood, but it is believed to involve a combination of genetic predisposition, immune system dysregulation, and environmental factors, such as smoking, obesity, and hormonal influences [6,7,8,9]. Genetic studies have shown variations in immune response and skin barrier function genes, while immune dysregulation and microbial dysbiosis within lesions contribute to the persistent inflammation [10,11,12,13,14]. The presence of a polymicrobial community, including antibiotic-resistant bacteria, complicates treatment and contributes to disease chronicity [15,16,17,18,19,20,21,22,23].

Antimicrobial resistance (AMR) among these bacterial populations poses substantial treatment challenges. Understanding AMR patterns in HS lesions is crucial for optimizing treatment strategies and improving clinical outcomes. This study aims to investigate the antimicrobial resistance profiles of bacterial isolates from HS lesions in a tertiary referral hospital in Greece to provide localized insights into resistance patterns and their treatment implications. Localized research is vital, as resistance patterns can vary by region and over time, influencing tailored therapeutic approaches and enhancing the quality of life for HS patients.

## 2. Materials and Methods

Following institutional review board clearance, this prospective study enrolled consecutive adult patients with hidradenitis suppurativa (HS) who visited the Dermatology Department at the University Hospital in Greece between January 2019 and December 2023. Eligible patients were those who were aged 18 years or older, who had not received topical or systemic antibiotic therapy in the preceding three months, and who presented with actively inflamed purulent HS lesions suitable for skin swab collection. Various enriched, selective, and differential culture media (all from bioMérieux SA) were employed to optimize the isolations of both non-fastidious and fastidious microorganisms from clinical samples obtained from HS lesions. These media were selected based on their ability to support the growth of diverse bacterial species under specific incubation conditions tailored to enhance microbial recovery and identification.

In the laboratory setting, after swabs were inoculated onto appropriate agar plates, meticulous procedures were followed to ensure comprehensive processing of clinical samples. This included conducting wet mount preparations and Gram-stained smears, fundamental techniques that aid in the initial characterization and preliminary identification of microbial morphology and staining characteristics. Each step adhered to standard culture protocols to maintain consistency and reliability in microbial isolation and identification. The choice of culture media and incubation conditions was critical to fostering the growth of both aerobic and anaerobic bacteria, reflecting the polymicrobial nature commonly observed in HS lesions.

By employing a systematic approach in the laboratory, this study aimed to capture a broad spectrum of microbial diversity present in HS lesions, ranging from more common pathogens like *Staphylococcus aureus* to less frequently isolated anaerobic species. This comprehensive microbial profiling is essential for understanding the complex microbial community dynamics within HS lesions and its potential implications for disease severity and treatment outcomes.

Moreover, the use of standardized culture techniques and selective media ensured the robustness of the data generated, facilitating the accurate identification of bacterial species involved in HS pathogenesis. These methodologies are pivotal in advancing our knowledge of antimicrobial resistance patterns and guiding effective therapeutic interventions tailored to combat the challenges posed by multidrug-resistant organisms in HS management.

Bacterial identification in our study utilized a combination of standard biochemical assays and the VITEK 2 automated system, supplemented by confirmation through matrix-assisted laser desorption/ionization time-of-flight mass spectrometry (MALDI-TOF MS) version 3.2 from bioMérieux SA [24,25,26]. This comprehensive approach ensured accurate species determination and reliable data for subsequent analyses.

Antibiotic susceptibility testing was conducted using the VITEK 2 automated system, adhering to the 2023 European Committee on Antimicrobial Susceptibility Testing (EUCAST) guidelines [25]. This standardized method allowed for a consistent interpretation of susceptibility profiles across all bacterial isolates, which is crucial for assessing their response to antibiotic treatments.

The term “antibiotic resistance” in our study encompassed acquired resistance, denoting bacteria classified as resistant or intermediately susceptible based on antibiogram profiles, and intrinsic resistance, which referred to bacteria inherently unaffected by specific antibiotics. Considering bacteria classified as intermediately susceptible alongside resistant strains was necessary, due to their potential to develop full resistance under prolonged antibiotic exposure, highlighting the importance of vigilant monitoring and tailored treatment strategies.

This rigorous approach to bacterial identification and antibiotic susceptibility testing provided a robust foundation for evaluating antimicrobial resistance patterns among isolates from hidradenitis suppurativa (HS) lesions. It underscored the complexity of managing infections in HS and emphasized the critical need for effective antimicrobial stewardship to combat rising resistance rates effectively.

Data analysis involved descriptive statistics and relative frequencies computed using IBM SPSS Statistics 25 [27], facilitating a comprehensive analysis of antimicrobial resistance patterns among the isolated bacterial strains from HS lesions.

This methodological framework ensures the rigorous collection, processing, and analysis of clinical samples, providing critical insights into antimicrobial resistance trends specific to HS. The study’s systematic approach enhances the understanding of microbial dynamics in HS pathogenesis and informs evidence-based clinical decision-making for optimized treatment strategies.

## 3. Results

The comprehensive data collected between January 2019 and December 2023 provided a detailed snapshot of the demographic, clinical, and bacteriological profiles of 103 patients with hidradenitis suppurativa (HS) who participated in this study. Among them, the distribution was 57.3% females and 42.7% males, with a median age of 35 years ± 12.96, reflecting a diverse cohort representative of the HS population. The staging of HS lesions varied, with 27.2% classified as Hurley Stage I, 50.5% as Hurley Stage II, and 22.3% as Hurley Stage III, highlighting the spectrum of disease severity observed in the patient group (Table 1).

Skin swabs collected from multiple anatomical sites revealed distinct microbial colonization patterns. Notably, 35.9% of samples originated from the inguinal area, 21.4% originated from the gluteal area, 29.1% originated from the axillary area, and 13.6% originated from the perianal area, underscoring the multifocal nature of HS lesions and the necessity for localized microbial analysis.

From the 139 purulent HS lesion swab samples analyzed, 79.9% tested positive for bacterial growth, highlighting the predominance of microbial involvement in HS pathogenesis. Gram-positive bacteria accounted for the majority of isolates (73%), with significant representation from families such as *Staphylococcaceae*, *Enterobacteriaceae*, and *Streptococcaceae*. *Staphylococcus epidermidis* emerged as the most prevalent species (16.7%), followed by *Staphylococcus haemolyticus*, *Staphylococcus lugdunensis*, *Proteus mirabilis*, and *Staphylococcus aureus*, each playing distinct roles in the microbial ecology of HS lesions (Table 2).

Antibiogram profiles revealed concerning levels of resistance among isolated bacterial species. For instance, *Staphylococcus lugdunensis* exhibited high resistance rates to levofloxacin (57.1%) and penicillin (53.3%), while *Staphylococcus aureus* displayed notable resistances to penicillin (76.9%) and fusidic acid (58.3%) (Table 3).

Gram-negative isolates also demonstrated significant resistance, with rates as high as 63.5% for tigecycline, 63.3% for ampicillin, and 40.5% for colistin (Table 4).

Anaerobic bacteria were similarly resistant, with rates ranging from 53.8% to 62.5% for erythromycin, clindamycin, and penicillin (Table 5).

Analyzing the distribution of bacterial isolates across anatomical regions further highlighted localized variations. In the inguinal area, anaerobes predominated (44.8%), followed by coagulase-negative *Staphylococcus* (32.8%), *Staphylococcus lugdunensis* (11.9%), and *Proteus mirabilis* (9%). Conversely, the axillary area predominantly hosted coagulase-negative staphylococci (34%), while the gluteal and perianal areas exhibited higher concentrations of anaerobic bacteria, aligning with previous studies emphasizing the roles of these bacteria in HS pathogenesis (Table 6).

These findings underscore the complexity of microbial colonization in HS and its implications for disease management. The detailed characterization of bacterial species and their resistance profiles provides critical insights for tailoring antimicrobial therapies, optimizing treatment outcomes, and addressing the challenges posed by multidrug-resistant organisms in HS management. This comprehensive dataset not only contributes to the understanding of HS pathophysiology but also informs strategies for antimicrobial stewardship and personalized treatment approaches in clinical practice.

## 4. Discussion

In this study, we prospectively analyzed the microbiological profiles of 103 consecutive adult HS patients from January 2019 to December 2023 who had not used topical or systemic antibiotics in the previous three months and presented with actively inflamed purulent HS lesions suitable for skin swab collection at a tertiary referral hospital in Greece. Of the 139 purulent HS lesion swab samples analyzed, 79.9% showed bacterial growth, emphasizing the significant role of microbes in HS pathogenesis. Gram-positive bacteria dominated the isolates (73%), with significant representation from families such as *Staphylococcaceae*, *Enterobacteriaceae*, and *Streptococcaceae*. The most common species identified was *Staphylococcus epidermidis* (16.7%), followed by *Staphylococcus haemolyticus*, *Staphylococcus lugdunensis*, *Proteus mirabilis*, and *Staphylococcus aureus*. Antibiogram profiles revealed high levels of resistance among the bacterial isolates. *Staphylococcus lugdunensis* showed high resistance rates to levofloxacin (57.1%) and penicillin (53.3%), while *Staphylococcus aureus* exhibited notable resistances to penicillin (76.9%) and fusidic acid (58.3%). Gram-negative isolates also demonstrated significant resistance, with rates of 63.5% for tigecycline, 63.3% for ampicillin, and 40.5% for colistin. Anaerobic bacteria showed resistance rates ranging from 53.8% to 62.5% for erythromycin, clindamycin, and penicillin. 

Our findings align with a previous study by Bettoli et al., which reported bacterial growth in 83.2% of samples collected from purulent HS lesions [20]. The most frequent bacteria identified in that study were *Proteus* spp. (13.5%), *E. coli* (9.8%), *S. epidermidis* (9.2%), and *S. agalactiae* (8.6%), suggesting that the invasion of commensal skin bacteria in HS lesions is likely a secondary event. Bettoli et al. also found that amoxicillin, oxacillin, erythromycin, and doxycycline were commonly associated with bacterial resistance. Clindamycin and rifampicin were resistant in 107 (65.6%) and 113 (69.3%) bacterial cultures out of the 163 strains isolated [20]. In our study population, tetracycline molecules, considered a valid alternative antibiotic for HS therapy, showed high resistance among bacterial isolates, with resistance in 138 (84.5%) cases.

According to the findings of our study, a systematic review highlighted the high prevalences of *S. aureus* and coagulase-negative staphylococci (CoNS) among various species isolated from the inflamed lesions of HS patients [28]. However, the review also included retrospective studies, which may have led to an underestimation of anaerobic bacteria due to lower identification rates. Additionally, bacterial identification methods varied significantly between 1988 and 2014, ranging from biochemical tests to MALDI-TOF mass spectrometry and molecular methods. Notably, *Actinomyces* spp. were not identified until the late 1990s.

The main goals of medical treatments for HS are to reduce inflammation and manage secondary infections; however, the impact that these medications have on the skin microbiota is rarely discussed. Antibiotics disrupt typical bacterial functions, such as the creation of proteins and cell walls, nucleic acid synthesis, membrane integrity, and metabolic pathways [11]. This disruption can significantly affect the microbial ecosystem of the skin, leading to both short-term and long-term changes in bacterial communities. Although there are insufficient randomized, placebo-controlled clinical trials to support the prudent use of antibiotics in the treatment of mild-to-severe HS, expert consensus guidelines based on case series do support this approach [29]. Topical antibacterial washes with chlorhexidine or benzoyl peroxide (BPO) and topical clindamycin are common antibacterial and antibiotic regimens for HS [30]. These topical treatments aim to reduce the bacterial load on the skin, thereby decreasing inflammation and the risk of secondary infections. Antibiotic therapy stands as a cornerstone in the management of hidradenitis suppurativa (HS), offering crucial relief from symptoms and aiding in the prevention of recurrence, thereby mitigating the risk of antibiotic resistance development [23]. This therapeutic approach is particularly beneficial in severe cases, where antibiotics serve not only to alleviate inflammation but also to prepare the affected area for subsequent surgical interventions by reducing infectious burdens [31,32,33,34].

Among the various antibiotic regimens employed, combination therapy featuring rifampicin and clindamycin has emerged as a preferred choice, due to its favorable outcomes in numerous HS patients [29,35,36,37]. Rifampicin and clindamycin combination therapy is a well-documented treatment approach for moderate-to-severe HS. This combination leverages the synergistic antibacterial effects of both drugs and their ability to reduce inflammation. A randomized controlled trial showed that the combination of oral clindamycin (300 mg twice daily) and rifampicin (300 mg twice daily) for 10 weeks was effective in treating moderate-to-severe HS [30]. The study reported significant improvements in clinical symptoms and quality of life for patients on this regimen. Oral clindamycin (usually 300 mg twice daily) can be prescribed for a more extensive disease [38,39]. It is less commonly used as monotherapy for moderate-to-severe HS, compared to combination therapy, due to concerns about antibiotic resistance and lower efficacy as a single agent. In another study, adalimumab initiated with clindamycin and rifampicin showed greater clinical effectiveness than adalimumab monotherapy [34,40]. An important difference in effect was observed in the decrease of draining tunnels, addressing a serious limitation of adalimumab monotherapy.

Research into the microbiomes of HS lesions has revealed significant dysbiosis compared to healthy skin, underscoring the pivotal role of bacterial communities in perpetuating the inflammatory cycle characteristic of HS [32,40,41,42,43,44]. Dysbiosis not only triggers inflammatory responses but also presents molecular targets that influence immune system interactions, further complicating disease management [45,46,47,48,49,50]. The evolving field of metagenomics holds promise in elucidating these intricate microbial ecosystems, potentially supplanting traditional culture-dependent methods in identifying pathogenic bacterial colonization in HS and other skin disorders [14,15,16,17].

While current research primarily focuses on direct bacterial identification, there remains a critical gap in understanding the antibiotic susceptibility profiles of these identified pathogens [19,20,21,22]. Conventional culture-dependent techniques, though readily available and widely used in hospital settings, have inherent limitations when compared to the comprehensive insights offered by metagenomic approaches.

In clinical practice, the judicious use of antibiotics involves selecting agents that specifically target isolated bacterial strains, thereby optimizing treatment efficacy while minimizing the emergence of resistance [34]. However, it is crucial for healthcare providers to balance the antimicrobial benefits with the potential risks to the patient’s commensal flora, which may lead to the development of antibiotic resistance in non-targeted organisms. As research continues to advance, bridging these gaps in knowledge will be essential for refining treatment protocols and enhancing therapeutic outcomes in HS management. By integrating insights from microbiome research and leveraging technological advancements in microbial analysis, clinicians can adopt more precise and effective strategies tailored to the individual needs of HS patients, ultimately improving long-term prognosis and quality of life.

Systemic antibiotics are also a mainstay in the treatment of HS. Tetracyclines, dapsone, IV ertapenem, and combination therapy involving clindamycin and rifampicin or moxifloxacin, metronidazole, and rifampicin are frequently used [38,39]. Many antibiotics have anti-inflammatory qualities in addition to their bacteriostatic or bactericidal properties. For instance, tetracyclines are known to decrease immune cell chemotaxis and reduce matrix metalloproteinase (MMP) activity, which likely contributes to their effectiveness in treating HS. Marasca and colleagues recently examined the pharmacologies, mechanisms of action, and efficacies of common HS antibiotic treatments, shedding light on their multifaceted roles in disease management [50]. Although data regarding these drugs’ effects on human microbial communities are known, their effects on the HS skin microbiome have not been directly evaluated. Antibiotics reduce the diversity and total burden of bacteria, potentially eliminating the microbial stimuli that trigger the host immune response. However, this reduction can also lead to an imbalance in the skin microbiota, known as dysbiosis, which may have implications for disease progression and recurrence. Targeted antibiotic therapy can completely destroy particular microbial communities (specific genera), but there is a chance that other community members may suffer unintended harm. Furthermore, it is uncertain how these communities will recover and what long-term effects these changed community structures (composition and function) will have. Ferrer and colleagues thoroughly reviewed the comparative effectiveness of 68 antibiotic regimens, either in isolation or in combination, on the abundance of particular microbial clades [48]. They found that some genera—*Bacteroides*, *Prevotella*, *Cutibacterium*, *Streptococcus*, *Staphylococcus*, *Corynebacterium*, and *Finegoldia*—are more sensitive to antibiotic manipulation than others, with some genera known to be altered in HS skin. Although they may not significantly change the microbial ecology, topical body washes may nonetheless be useful in reducing the microbial burden [34,41]. It is increasingly important to identify the microorganisms that are key components of the pathophysiology of HS, as epidemiological statistics show that rising antibiotic resistance closely follows increases in the number of prescriptions and the use of antibiotics. Careful elimination or reduction of the microbial culprits, with minimal collateral damage to skin commensals and without contributing to the rise in antibiotic resistance, is essential. Developing strategies that balance effective microbial management with the preservation of beneficial skin microbiota will be crucial in the future management of HS. This includes ongoing research into the specific roles of different microbial species in HS and the development of new therapeutic approaches that minimize the risk of antibiotic resistance. Despite the study’s limitations, such as the absence of a control group and potential selection bias, it provides valuable insights into the antimicrobial resistance profiles of bacterial isolates from HS lesions. These insights are crucial, as they highlight the urgent need for ongoing surveillance efforts and further research to guide informed clinical decision-making and enhance antimicrobial stewardship practices in HS management.

The study underscores the importance of continuously monitoring antimicrobial resistance trends among the bacteria commonly found in HS lesions. Regular surveillance can help detect emerging resistance patterns early, enabling clinicians to adjust treatment regimens accordingly and avoid the use of ineffective antibiotics. This proactive approach can help mitigate the spread of resistant strains and improve patient outcomes.

This study has several limitations. First, it was conducted in a single location at a university hospital. Since antimicrobial resistance patterns can vary significantly across different regions, the findings may not be generalizable to other areas, limiting the applicability of these results to broader populations. Second, the study enrolled 103 patients, which, while providing valuable insights, may not be large enough to capture the full diversity of HS patients. A larger sample size might have revealed additional patterns and resistance profiles. Third, the study was conducted over five years. While this allows for some temporal analysis, resistance patterns can evolve rapidly. Continuous surveillance beyond the study period is necessary to monitor ongoing changes in antimicrobial resistance. Fourth, patients who had received topical or systemic antibiotic therapy in the preceding three months were excluded. While essential to prevent skewing results, this criterion may limit the study’s applicability to all HS patients, particularly those recently treated for infections. Fifth, the study primarily focused on bacterial isolates and did not account for other potential pathogens, such as fungi or viruses, that might play a role in HS lesions. This limits the comprehensiveness of the microbial profile associated with HS. Sixth, while the study provides a snapshot of resistance patterns, it lacks longitudinal data to observe how individual patients’ resistance profiles and treatment responses evolve. Lastly, despite the rigorous approach, there is always a possibility of methodological biases, such as sample collection and processing inconsistencies, which could affect the accuracy and reliability of the results. Addressing these limitations in future research could enhance the understanding of antimicrobial resistance in HS and improve the generalizability and applicability of the findings.

Further research is essential to deepen our understanding of the mechanisms driving antimicrobial resistance in HS. Investigating the genetic and molecular bases of resistance can reveal potential targets for new antimicrobial agents. Studies focusing on the microbiome of HS lesions and its interaction with host immune responses could also provide insights into novel therapeutic strategies. For instance, understanding how dysbiosis contributes to inflammation and infection in HS may lead to the development of treatments that restore a healthy microbiota balance, reducing the reliance on antibiotics.

Developing new antimicrobial agents is a critical step in addressing the escalating threat of antimicrobial resistance in the context of HS. Innovative antibiotics that target specific resistance mechanisms or have novel modes of action can offer new options for treating resistant infections. Additionally, exploring alternative treatment modalities, such as bacteriophage therapy, antimicrobial peptides, and immunomodulatory therapies, can provide complementary or substitute approaches to traditional antibiotics.

Moreover, incorporating antimicrobial stewardship principles into HS management practices is essential. This involves not only choosing the right antibiotic based on susceptibility profiles but also optimizing the duration and dosage to minimize resistance development. Educating healthcare providers and patients about the importance of responsible antibiotic use can further support these efforts.

In conclusion, while the study has its limitations, it makes a significant contribution to our understanding of antimicrobial resistance in HS. It emphasizes the need for ongoing surveillance, further research, and the development of new therapeutic strategies to combat resistance. By addressing these challenges, we can improve the management of HS and safeguard the effectiveness of antibiotics for future generations.

## Figures and Tables

**Table 1:** Bacteriology, demographic, and clinical characteristics of the 103 patients with hidradenitis suppurativa.

Number of patients included	103
Number of swab samples	139
Number of positive swab samples	111/139 (79.9%)
Number of bacterial growths	175
Gram-positive	128/175 (73.1%)
Gram-negative	47/175 (26.9%)
Aerobes	149/175 (85.1%)
Anaerobes	26/175 (14.9%)
Sampled areas	
Inguinal	37/103 (35.9%)
Gluteal	22/103 (21.4%)
Axillary	30/103 (29.1%)
Perianal	14/103 (13.6%)
Age/years (median ± SD)	35 ± 12.96
Age range	17–67
Gender	
Male	44/103 (42.7%)
Female	59/103 (57.3%)
Hurley stage	
Hurley stage I	28/103 (27.2%)
Hurley stage II	52/103 (50.5%)
Hurley stage III	23/103 (22.3%)
Smoking status	
Current smokers	68/103 (66%)
Non-smokers	19/103 (18.4%)
Ex-smokers	16/103 (15.5%)

SD: standard deviation.

**Table 2:** Microbiological characteristics of the pathogens isolated from patients’ samples.

	Isolates	No.
**AEROBES**	**Gram-positives**	
	*Staphylococcus aureus*	13
	*Staphylococcus lugdunensis*	15
	*Staphylococcus* spp.	56
	*Streptococcus* spp.	13
	*Enterococcus faecalis*	7
	*Corynebacterium* spp.	8
	**Gram-negatives**	
	*Proteus mirabilis*	14
	*Escherichia coli*	6
	*Citrobacter koseri*	3
	*Klebsiella* spp.	4
	*Enterobacter cloacae*	1
	*Serratia marcescens*	1
	*Raoultella ornithinolytica*	1
	*Acinetobacter* spp.	4
	*Pseudomonas aeruginosa*	2
	*Burkhoderia cepacia*	1
**ANAEROBES**	**Gram-positives**	
	*Peptoniphilus* spp.	1
	*Peptostreptococcus anaerobius*	2
	*Finegoldia magna*	3
	*Anaerococcus* spp.	2
	*Actinomyces* spp.	2
	*Actinotignum schaalii*	1
	*Schaalia radingae*	1
	*Bifidobacterium* spp.	1
	*Cutibacterium* spp.	2
	Other Gram-positive bacillus	1
	**Gram-negatives**	
	*Bacteroides* spp.	2
	*Prevotella* spp.	5
	*Fusobacterium* spp.	2
	*Veillonella* spp.	1

**Table 3:** In vitro activities of 16 antimicrobial agents tested against *Staphylococcus lugdunensis*, *Staphylococcus aureus*, and other coagulase-negative staphylococci isolates from the lesions of 103 patients with hidradenitis suppurativa.

Antibiotics	*S. aureus* (*n* = 13)		*S. lugdunensis* (*n* = 15)		Other Coagulase-Negative Staphylococci (*n* = 56)	
	Susceptibility *n* (%)	Resistance *n* (%)	Susceptibility *n* (%)	Resistance *n* (%)	Susceptibility *n* (%)	Resistance *n* (%)
Penicillin	3/13 (23.1)	10/13 (76.9)	7/15 (46.7)	8/15 (53.3)	4/45 (8.9)	41/45 (91.1)
Oxacillin	7/13 (53.8)	6/13 (46.2)	13/15 (86.7)	2/15 (13.3)	19/55 (34.5)	36/55 (65.5)
Gentamicin	11/13 (84.6)	2/13 (15.4)	15/15 (100)	0/15 (0)	43/56 (76.8)	13/56 (23.2)
Levofloxacin	7/11 (63.6)	4/11 (36.4)	6/14 (42.9)	8/14 (57.1)	33/53 (62.3)	20/53 (37.7)
Erythromycin	9/13 (69.2)	4/13 (30.8)	12/15 (80)	3/15 (20)	7/56 (12.5)	49/56 (87.5)
Clindamycin	10/13 (76.9)	3/13 (23.1)	12/15 (80)	3/15 (20)	22/56 (41.1)	33/56 (58.9)
Linezolid	13/13 (100)	0/13 (0)	15/15 (100)	0/15 (0)	56/56 (100)	0/56 (0)
Daptomycin	12/12 (100)	0/12 (0)	15/15 (100)	0/15 (0)	50/50 (100)	0/50 (0)
Teicoplanin	13/13 (100)	0/13 (0)	13/15 (86.7)	2/15 (13.3)	50/53 (94.3)	3/53 (5.7)
Vancomycin	13/13 (100)	0/13 (0)	15/15 (100)	0/15 (0)	56/56 (100)	0/56 (0)
Tetracycline	11/13 (84.6)	2/13 (15.4)	14/14 (100)	0/14 (0)	31/56 (55.4)	25/56 (44.6)
Tigecycline	13/13 (100)	0/13 (0)	15/15 (100)	0/15 (0)	50/52 (96.2)	2/52 (3.8)
Fusidic Acid	5/12 (41.4)	7/12 (58.3)	13/15 (86.7)	2/15 (13.3)	7/56 (12.5)	49/56 (87.5)
Mupirocin	12/12 (100)	0/12 (0)	14/15 (93.3)	1/15 (6.7)	3/14 (21.4)	11/14 (78.6)
Rifampicin	13/13 (100)	0/13 (0)	15/15 (100)	0/15 (0)	54/56 (96.4)	2/56 (3.6)
Trimethoprim/sulfamethoxazole	13/13 (100)	0/13 (0)	15/15 (100)	0/15 (0)	45/56 (80.4)	11/56 (19.6)

*n*: number of isolates in which the antibiotic was tested.

**Table 4:** In vitro activities of 18 antimicrobial agents tested against 37 Gram-negative isolates from the lesions of 103 patients with hidradenitis suppurativa.

Antibiotic	S (%)	R (%)
Ampicillin	11/30 (36.7)	19/30 (63.3)
Amoxicillin/clavulanic acid	19/29 (65.5)	10/29 (34.5)
Cefuroxime	26/31 (83.9)	5/31 (16.1)
Cefotaxime	31/35 (88.6)	4/35 (11.4)
Ceftriaxone	29/32 (90.6)	3/32 (9.4)
Ceftazidime	33/35 (94.3)	2/35 (5.7)
Cefepime	34/34 (100)	-
Aztreonam	30/34 (88.2)	4/34 (11.8)
Imipenem	30/30 (100)	-
Meropenem	30/30 (100)	-
Amikacin	36/37 (92.3)	1/37 (2.7)
Gentamicin	35/37 (94.6)	2/37 (5.4)
Tobramycin	35/37 (94.6)	2/37 (5.4)
Colistin	22/37 (59.5)	15/37 (40.5)
Ciprofloxacin	14/15 (93.3)	1/15 (6.7)
Tigecycline	8/22 (36.4)	14/22 (63.6)
TMP/SMX	13/15 (86.7)	2/15 (13.3)
Fosfomycin	27/29 (93.1)	2/29 (6.9)

S, susceptible; R, resistant; TMP-SMX, trimethoprim/sulfamethoxazole.

**Table 5:** In vitro activities of 11 antimicrobial agents tested against 26 anaerobes isolated from the lesions of 103 patients with hidradenitis suppurativa.

Antibiotic	S (%)	R (%)
Penicillin	12/26 (46.2)	14/26 (53.8)
Ampicillin	19/26 (73.1)	7/26 (26.9)
Amoxicillin/clavulanic acid	24/26 (92.3)	2/26 (7.7)
Piperacillin/tazobactam	23/26 (88.5)	3/26 (11.5)
Cefoxitin	25/26 (96.2)	1/26 (3.8)
Erythromycin	9/24 (37.5)	15/24 (62.5)
Clindamycin	10/26 (38.5)	16/26 (61.5)
Chloramphenicol	26/26 (100)	0/26 (0)
Tetracycline	17/25 (68)	8/25 (32)
Metronidaxole	13/26 (50)	13/26 (50)
Vancomycin	16/24 (66.7)	8/24 (33.3)

S, susceptible; R, resistant.

**Table 6:** Topographic distribution of the 174 bacterial isolates from 103 patients with hidradenitis suppurativa (HS).

Bacteria	Axillary Area	Inguinal	Gluteal	Perianal	Total
*Staphylococcus lugdunensis*	1	8	5	1	15
*Staphylococcus aureus*	6	5	1	1	13
Coagulase-negative staphylococci	17	22	9	8	56
*Enterococcus faecalis*	3	2	2	0	7
*Proteus mirabilis*	4	6	4	0	14
*Escherichia coli*	3	2	1	0	6
*Pseudomonas aeruginosa*	1	1	0	0	2
Other aerobes	0	1	3	1	5
Anaerobes	15	20	10	11	56
Total	50	67	35	22	174

## Data Availability

The data presented in this study are available upon request from the corresponding author.
